# Supplementary material for: iPSC-Derived Hereditary Breast Cancer Model Reveals the BRCA1-Deleted Tumor Niche as a New Culprit in Disease Progression
Source: Int J Mol Sci. 2021 Jan 27;22(3):1227. doi: 10.3390/ijms22031227 (PMC7866119; doi:10.3390/ijms22031227)
Supplement: Supplementary file 1 [file ijms-22-01227-s001.pdf]

International Journal of
Molecular Sciences

MDPI

Supplementary Material

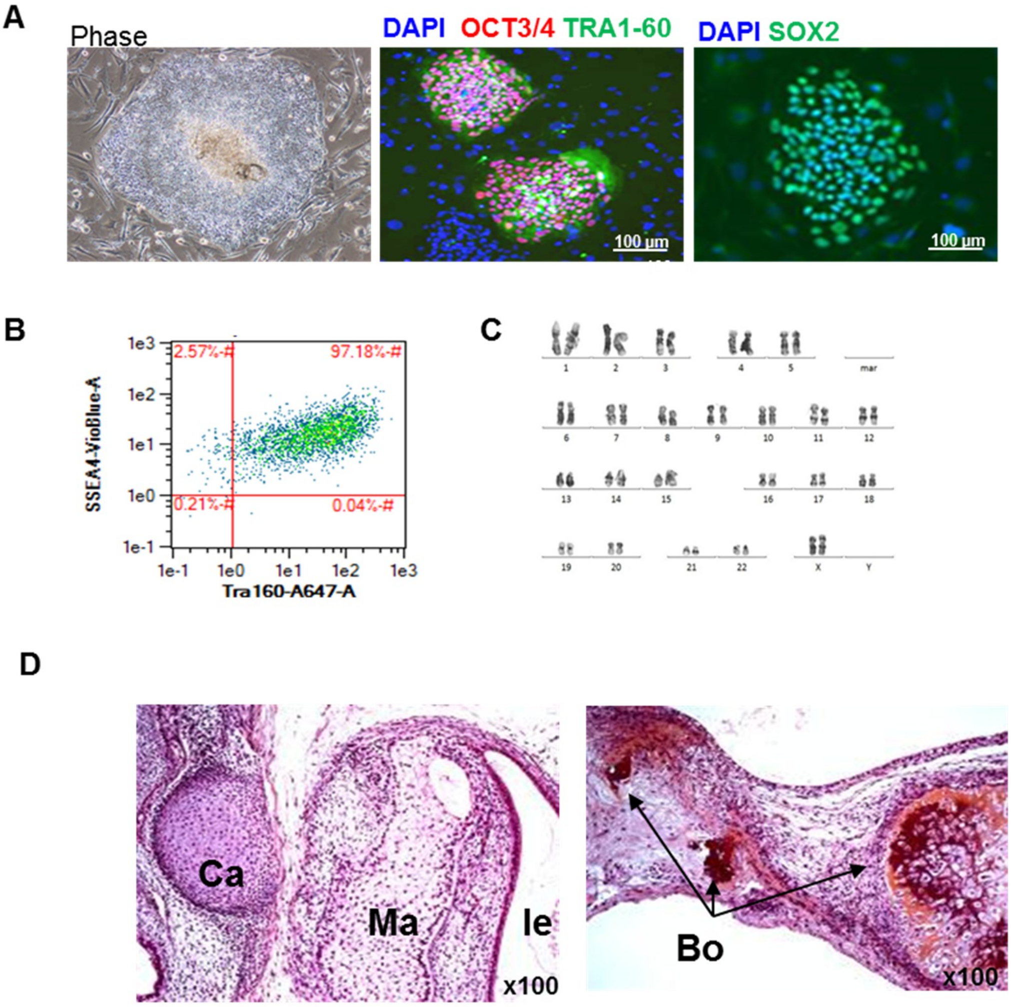

Figure S1. Characterization of the iPSC line with a deletion of exon17 of the *BRCA1* gene. (**A**) Phase contrast imaging of BRCA1del17 iPSC colonies (polyclonal stock) grown on MEF stromal cells at passage 60. iPSC colonies were positive for oct3/4, Tra1-60, and Sox2 by immunofluorescence staining. (**B**) FACS evaluation of pluripotency markers using double SSEA4 and TRA-1-60 staining, confirming the presence of cell surface pluripotency markers on BRCA1del17 iPSCs. (**C**) Cytogenetic analysis showing a normal karyotype (46,XX). (**D**) Pathological analysis of a teratoma from BRCA1del17 iPSCs, showing normal ectodermal, endodermal, and mesodermal differentiation Ca: Cartilage area, Ma: Malpighian epitheliums, Ie: Intestinal epithelium, Bo: Bone structure.

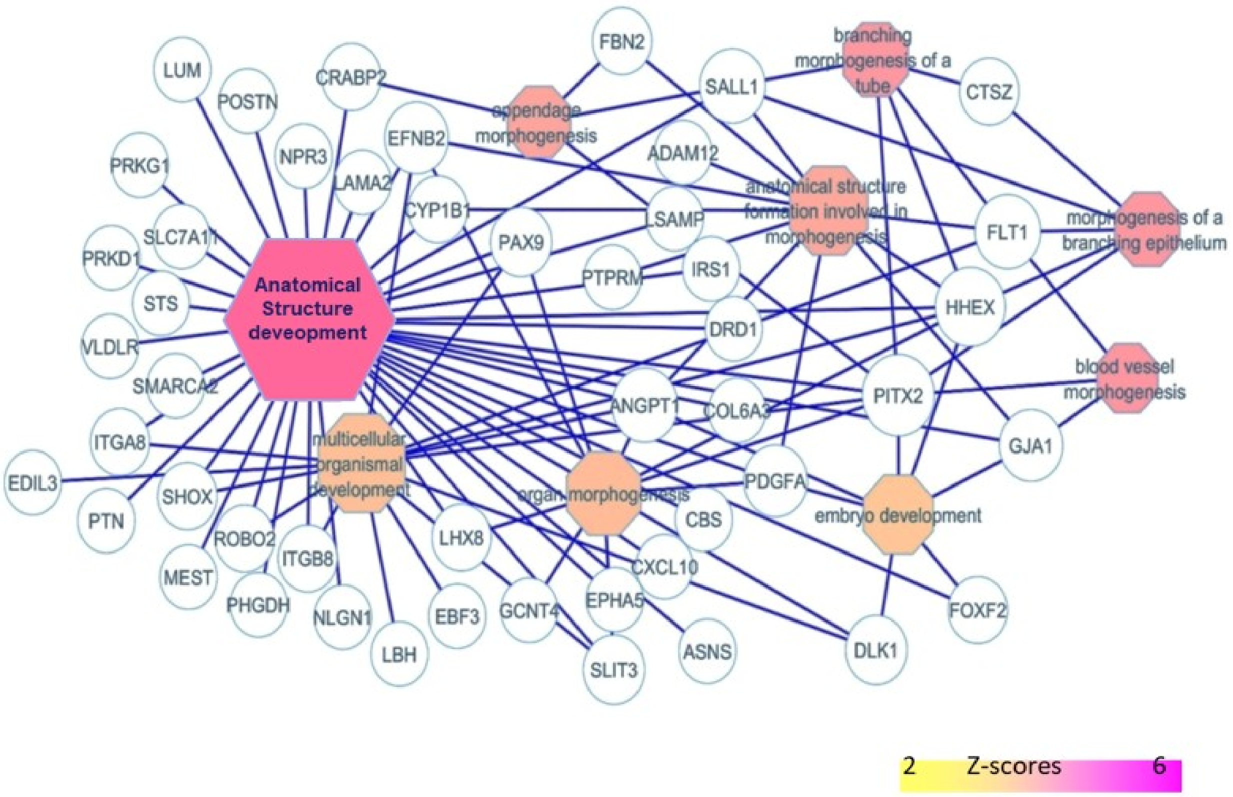

Figure S2. Functional enrichment in developmental functionalities of genes that were upregulated in *BRCA1*+/- iMSCs compared to *BRCA1*+/+ iMSCs.

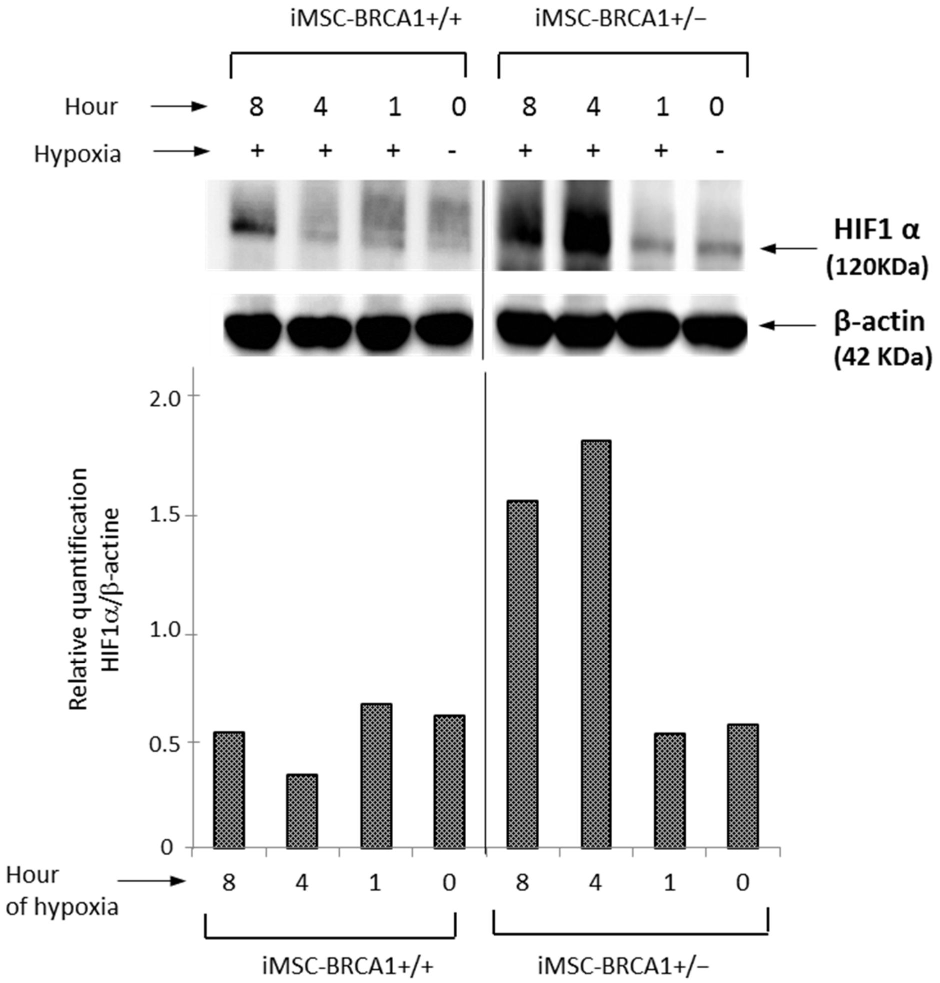

Figure S3. Detection of HIF-1α by western blot analysis. (**A**) Detection of HIF-1α levels in *BRCA1*+/- and *BRCA1*+/+ iMSCs at 1, 4, and 8 h of culture under hypoxia. (**B**) Quantification of HIF-1α levels relative to β-actin in *BRCA1*+/- compared to *BRCA1*+/+ iMSCs at 1, 4, and 8 h of culture under hypoxia.

angiogenesis gene	Gene ID NCBI	meanBRCA1+/- (log2 Expression)	mean BRCA1+/+ (log2 expression)	Fold Change BRCA1+/ - versus BRCA1+/+
LUM	4060	8.6374635	3.5491985	34.01
ANGPT1	284	7.5209795	2.710738	28.05
COL3A1	1281	9.5762815	5.5462315	16.33
PDGFRA	5156	8.5546665	5.477394	8.44
POSTN	10631	10.5032	7.8140715	6.44
EFNB2	1948	6.2215065	3.688202	5.78
PRKD1	5587	6.847697	4.3635915	5.59
FAP	2191	9.859882	7.412566	5.45
SEMA5A	9037	10.217915	7.859036	5.12
CYP1B1	1545	6.096288	4.056088	4.11
VCAN	1462	7.3576985	5.385929	3.92
MEIS1	4211	7.5872335	5.8451445	3.34
OLR1	4973	5.705447	3.9763295	3.31
TNFRSF21	27242	8.6978445	7.299534	2.63
GPR124	25960	7.5013175	6.103698	2.63
CDH13	1012	7.9806795	6.7465545	2.35
TEK	7010	6.395419	5.1983055	2.29
BMP4	652	5.024309	3.864698	2.23
MMP2	4313	11.87299	10.76546	2.15
PDGFA	5154	6.64465	5.568746	2.10
TCF21	6943	4.6996755	3.6841975	2.02
LRP5	4041	7.226323	6.2319165	1.99
HTATIP2	10553	6.2311365	5.262625	1.95
FOXC2	2303	6.7987355	5.838504	1.94
HOXB3	3213	5.815639	4.900051	1.88
PITX2	5308	5.625959	4.7189215	1.87
E2F7	144455	8.2231295	7.350838	1.83
NRP2	8828	7.3451265	6.488936	1.81
S100A4	6275	4.0790885	3.23863	1.79
WARS	7453	7.114368	6.3124075	1.74
MCAM	4162	6.1976745	5.4133865	1.72
ID1	3397	5.00556	4.2269865	1.71
NOV	4856	4.640248	3.9169345	1.65
ADM2	79924	5.8347425	5.148555	1.60
EPAS1	2034	9.438982	8.8155675	1.54
PDGFRB	5159	8.0641325	7.4521405	1.52

VEGFB	7423	6.52653	5.9486795	1.49
CTGF	1490	11.40989	10.841415	1.48
EDNRA	1909	5.4007295	4.83582	1.47
GREM1	26585	11.71062	11.147535	1.477
VASH1	22846	5.235575	4.6725475	1.47
ANG	283	4.721105	4.1803975	1.45
VEZF1	7716	8.218696	7.693095	1.43
MSX1	4487	5.760676	5.2415385	1.43
SPP1	6696	5.018289	4.51328	1.41
XBP1	7494	7.9175735	7.4259615	1.40
PLCD1	5333	5.2616355	4.77339	1.40
NCL	4691	7.6303435	7.190793	1.35
PARVA	55742	9.6359415	9.235568	1.31
COL18A1	80781	6.0921055	5.702126	1.31
EPHB3	2049	4.24314	3.86051	1.30
ELK3	2004	7.6747345	7.3024745	1.29
RNF213	57674	8.00176	7.6327015	1.29
KLF5	688	5.330473	4.99116	1.26
TNFAIP2	7127	6.737656	6.4018	1.26
ROBO1	6091	8.028958	7.6946285	1.26
NOTCH1	4851	6.40107	6.0752545	1.25
THBS1	7057	12.42167	12.101085	1.24
GPI	2821	9.3627525	9.0432905	1.24
LOXL2	4017	10.64943	10.330035	1.24
C1GALT1	56913	8.429931	8.1141335	1.24
ACVR1	90	7.504516	7.189317	1.24

Table S1. Angiogenesis related genes found overexpressed in MSCs BRCA1+/- versus MSCs BRCA1+/+: for each angiogenesis related genes provided in rows, columns from left to right describe respective gene symbol, NCBI gene identifier number, microarray logarithm base 2 of expression mean in MSCs BRCA1+/- samples, microarray logarithm base 2 of expression mean in MSCs BRCA1+/+ samples and fold change of expression between MSCs BRCA1+/- samples and MSCs BRCA1+/+ samples.

	Normoxia		Hypoxia	
	MSC BRCA1+/+	MSC BRCA1+/-	MSC BRCA1+/+	MSC BRCA1+/-
PDGFa	1	2,09	1	2,11
PDGFb	1	2,66	1	0,8
PDGFRa	1	4,32	1	2,31
PDGFRb	1	2,35	1	1,75
KDR	1	1,24	1	0,59
Flt1	1	0,68	1	2,61
VEGFa	1	1,15	1	1,13
VEGFb	1	1,08	1	0,85
VEGFc	1	2,5	1	2.00
VEGFd	1	0,86	1	0,71
TEK	1	4,17	1	1,79
Angpt1	1	3,05	1	1,66
Angpt2	1	1,16	1	1,8

Table S2. RT-PCR data normalized to the control (MSC BRCA1+/+) for which values are set at 1, both under normoxia and hypoxia conditions.
